# Spontaneous Perspective Taking in Humans?

**DOI:** 10.3390/vision1020017

**Published:** 2017-06-16

**Authors:** Geoff G. Cole, Mark A. Atkinson, Antonia D. C. D’Souza, Daniel T. Smith

**Affiliations:** 1 Centre for Brain Science, University of Essex, Colchester CO4 3SQ, UK; 2 Department of Psychology, University of Exeter, Exeter EX4 4QJ, UK; 3 Department of Psychology, University of Durham, Durham DH1 3HP, UK

**Keywords:** perspective taking, vision, theory of mind, social attention

## Abstract

A number of social cognition studies posit that humans spontaneously compute the viewpoint of other individuals. This is based on experiments showing that responses are shorter when a human agent, located in a visual display, can see the stimuli relevant to the observer’s task. Similarly, responses are slower when the agent cannot see the task-relevant stimuli. We tested the spontaneous perspective taking theory by incorporating it within two classic visual cognition paradigms (i.e., the flanker effect and the Simon effect), as well as reassessing its role in the gaze cueing effect. Results showed that these phenomena (e.g., the Simon effect) are not modulated according to whether a gazing agent can see the critical stimuli or not. We also examined the claim that previous results attributed to spontaneous perspective taking are due to the gazing agent’s ability to shift attention laterally. Results found no evidence of this. Overall, these data challenge both the spontaneous perspective taking theory, as well as the attentional shift hypothesis.

## 1. Introduction

The ability to attribute mental states (e.g., desires, beliefs) to other individuals (‘Theory of Mind’, ToM) is often assumed to be central to efficient social cognition. Indeed, not knowing what others know can lead to difficulty when interacting socially. A number of studies (e.g., [1,2]) have suggested that one particular ToM attribute can occur automatically such that it is rapid and does not require controlled processing. Specifically, these authors posit that humans spontaneously represent the viewpoint or visual perspective of other individuals.

This notion has primarily found support from results obtained in the “dot-perspective” paradigm ([1]; see also [3]). In the basic procedure, participants are shown an image of a human avatar positioned in the centre of a virtual room that looks towards a left or right-hand wall. Positioned on the walls are a variable number of dots. For instance, there may be one on the left and two on the right, none on the left and one on the right, or one on each wall. Participants are required to rapidly judge how many dots are present from either their own perspective or that of the avatar. The important manipulation is whether the avatar and participant can see the same number of dots or a different number. For example, when the room contains two dots (only) located on the left wall and the avatar looks towards the left, both the participant and the avatar see the same number (i.e., two). If, however, an additional dot is added to the righthand wall, the participant and avatar now see a different number of dots; the avatar sees the two on the left she is facing, but the participant can see all three. Results typically show that reaction time (RT) to determine the number of dots is shorter when the participant and avatar can see the same number of dots compared to when they see a different number. Importantly, this occurs when participants are asked to perform the dot-number judgment from their own perspective, i.e., when the avatar’s perspective is irrelevant. It is these results that have led researchers to suggest that the perspective of another individual is spontaneously computed by an observer. 

Samson and colleagues [1] also suggested that the mechanisms responsible for the classic gaze cueing effect [4,5], in which attention is shifted to a gazed-at location, could provide a mechanism with which involuntary perspective taking occurs. As Samson et al. stated, “It is likely that similar attention cueing effects produced by the avatar’s gaze, head, and/or body orientation contributed to the ease with which the avatar’s visual experience was computed” [1] (p. 1264). Indeed, if the avatar acts as a directional cue, this would facilitate responses when the only discs in the display are located on the wall looked at by the avatar. By contrast, responses would be slowed when a disc appears on the wall not looked at because now attention would have to reorient away from the gazed-at wall to the other side of the display. However, rather than being seen as a mechanism, any attentional shift induced by the avatar can be considered a confound; Samson and colleagues’ [1] ‘consistency’ manipulation (of the avatar and participant’s viewpoint) maps directly on to the ‘validity’ manipulation used in standard gaze cueing experiments. In other words, a reflexive shift of attention could explain Samson et al.’s [1] data without any mentalistic attribution processes taking place. This alternative “directional hypothesis” has recently been examined by Santiesteban et al. [6]. The authors suggested “that it is the directional, rather than the agentive, features of the avatar that are important, and that they modulate a process that represents the number of dots on one side of the screen, rather than the number that an agent can see”([6], p. 930). Santiesteban and colleagues [6] go on to suggest that the shift of attention is induced by the ‘front features’ of the avatar such as the forehead, eyes, and nose. In support of this assertion, the authors presented participants with two sets of trial types in which either the avatar or an arrow was shown in the centre of the display. They found that both stimuli resulted in a consistency/cueing effect. Since an arrow cannot have a perspective or mental state, the authors suggested that results from the dot-perspective task do not show spontaneous perspective taking. Rather, the authors argued, the consistency effect is due to a domain-general process that facilitates the representation of one side of the display, the side gazed towards by the avatar. However, the demonstration that arrows generate a consistency effect does not falsify the claim that spontaneous perspective taking also generates a consistency effect. Put simply, showing that avatars can shift attention will not tell us anything about whether their perspective is taken. This can be no more true than saying that we take the arrow’s perspective in an arrow cueing experiment. Indeed, Santiesteban et al. [6] effectively argued that a replication of the classic central/arrow cueing effect challenges the spontaneous perspective taking hypothesis. Cole et al., ([7]; see also [8]) did attempt to falsify the theory that humans spontaneously compute the perspective of others by placing a physical barrier between the gazing agent and a target. Cole et al. adopted this barrier technique from work examining whether chimpanzees know what another individual can see (e.g., [9]). Clearly, when this method is employed in the dot-perspective paradigm, no perspective taking-like effect should be observed because the agent cannot see the same thing as the participant. However, Cole et al. [7] found the same dot-perspective-like data irrespective of whether the avatar could see the dots or not. A related problem for the attentional shift account (although not the spontaneous perspective taking theory) is that any shift induced by the avatar could itself be due to visual perspective taking. Indeed, a number of authors have argued that the classic gaze cuing effect can itself be modulated according to what the gazing agent can see. For instance, Nuku and Bekkering ([3]; see also [10,11]) showed that the size of the gaze cueing effect was smaller when the gaze cue had its eyes closed versus open, or when its vision was blocked out by a dark rectangle versus wearing sunglasses.

The principal aim of the present work was twofold. First, we tested the perspective taking theory by using two different effects as dependent measures. In Experiment 1 observers undertook a classic flanker task [12], and in Experiment 2 they performed a Simon task [13]. The spontaneous perspective taking theory predicts that the effects of flankers and spatial compatibility should be modulated according to whether an avatar can see the stimuli that induce these effects or not. In Experiment 3, the avatar was replaced by a gazing agent typical of those used in gaze following experiments and we again manipulated what the agent could see via a physical barrier. This was motivated by the contrasting findings of previous studies in which some report that the perspective of a gaze cue influences gaze following but others do not. Our second aim was to examine the attentional cueing account of Samson et al. [1] and Santiesteban et al. [6]. In Experiments 4 and 5, therefore, we employed the Samson et al. [1] avatars and assessed whether these stimuli are able to shift an observer’s attention laterally as these authors have suggested.

### 1.1. Experiment 1 Introduction

The implicit assumption of the ‘mentalising’ account proposed by Samson et al. [1] is that the RT cost on inconsistent trials occurs because there is interference between the internal representation of the number of dots the participant can see, and the internal representation of the number of dots the avatar can see. It is, therefore, reasonable to assume that this should manifest itself in other paradigms in which, crucially, the avatar either sees the same stimuli as the participant or does not. To this end, Experiment 1 required participants to perform a task based on the ‘flanker’ effect. It is well established that when a central letter has to be discriminated, flanking letters influence RT [12]. The flanker effect is particularly pronounced on the letter congruency version of the task in which the identity of a flanking letter may be different to the target but is part of the participant’s current response set, meaning that it is sometimes a target itself (on other trials). For instance, a participant may be asked to press a left-hand button when the target is an ‘A’ and a right-hand button when the target is a ‘B’. RTs are particularly slow if the target is an ‘A’ and a flanking letter is a ‘B’. Conversely, RTs are particularly fast if both the target and the distractor are the same.

**Figure 1:**
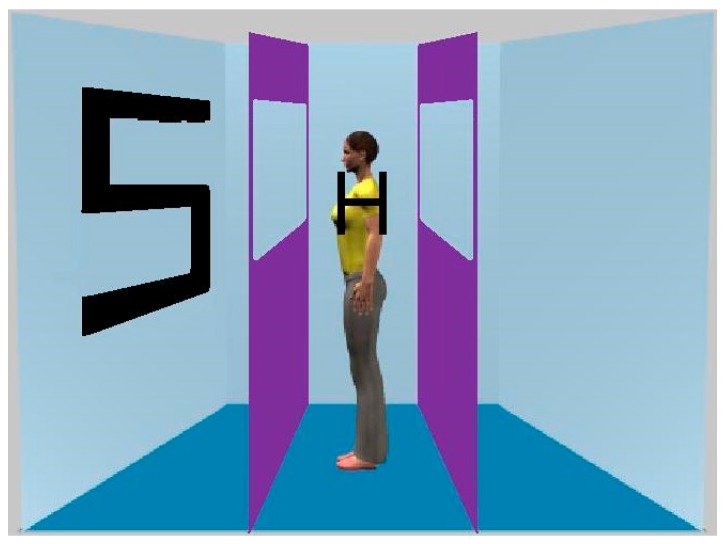
A stimulus used in Experiment 1. Note that in order to have parity with our other experiments, the barriers were also present, but always allowed the avatar to see the lateral wall.

In the present experiment, observers were required to determine the identity of a central letter in the presence of a single peripheral letter. The target was positioned on the shoulder of an avatar located in the centre of the room (see Figure 1). Crucially, either the avatar faced towards the flanking letter or faced towards the opposite wall. The rationale for this manipulation is that if the perspective of the avatar is spontaneously computed, the flanker effect should be magnified when the avatar is looking at the distractor relative to when the avatar is looking away from the distractor. 

#### Experiment 1 Results

**Figure 2:**
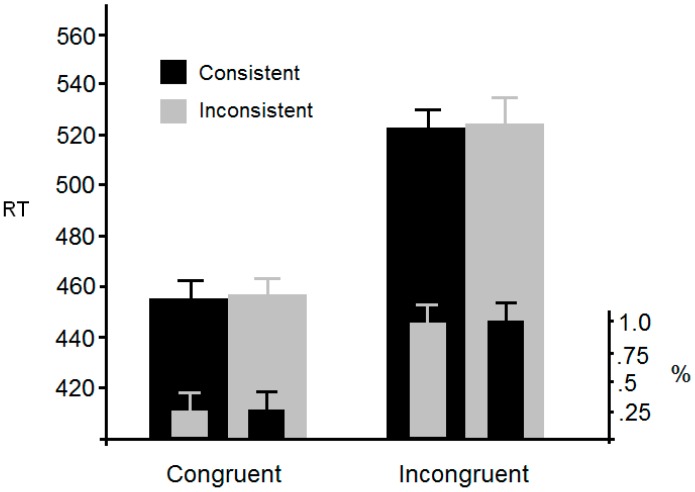
Mean RT (reaction time) and error rates from Experiment 1 together with standard errors. Consistent/Inconsistent refers to the location of the flanker with respect to where the avatar was looking. Congruent/Incongruent refers to the identity of the flanker with respect to the target letter, i.e., the manipulation that generates the classic flanker effect.

Figure 2 (overleaf) shows mean RTs for each of the four conditions. Outliers (2 SDs) accounted for 4.8% of responses and were omitted from further analysis. An ANOVA with congruency and consistency as within-participants factors revealed a significant main effect of congruency, *F*(1, 25) = 374, *p* < 0.0001, η^2^_p_ = 0.94, but no significant main effect of consistency, *F*(1, 25) < 1. The interaction was not significant, *F*(1, 25) < 1. Analysis of the error data using the same factors and levels revealed a significant main effect of congruency, *F*(1, 25) = 27.2, *p* < 0.001, η^2^_p_ = 0.52, but no significant main effect of consistency, *F*(1, 25) < 1. The interaction was also not significant, *F*(1, 25) < 1. 

Experiment 1 has revealed a classic flanker effect; RTs and accuracy were compromised when a peripheral (distractor) letter was incongruent with a central target. However, this effect was no smaller when the avatar faced away from the distracting element compared to when it faced towards it. This result does not, therefore, support the view that another person’s perspective is spontaneously taken. At best, these results suggest that the spontaneous perspective taking effect is not sufficiently reliable to generalise beyond the canonical perspective taking task when the stimuli and/or task are slightly modified.

### 1.2. Experiment 2 Introduction

**Figure 3:**
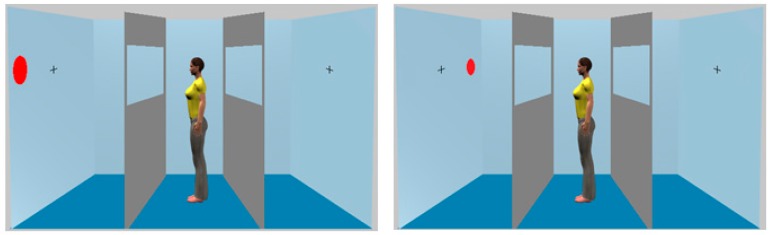
The stimuli employed in Experiment 2. In the left panel, the target is on the left side for both the participant and the avatar. By contrast, in the right panel the target is on the left side for the participant but on the right side for the avatar. Note that we again placed barriers either side of the avatar but the avatar could always see the targets.

One of the most robust phenomena of visual cognition is the finding that RTs are reduced when the stimulus to be responded to shares a spatial property with the effector used to respond to that stimulus (the stimulus/response compatibility, or ‘Simon effect’; e.g., [13]). For instance, responses to a target requiring a left-hand button press will be quicker if the target occurs on the left side of a display as opposed to the right. The stimulus location (i.e., left and right) is, of course, relative to the viewpoint of the observer. However, if observers spontaneously take the perspective of other individuals, then the representation of a stimulus location should be affected by the viewpoint of that individual. For instance, a stimulus located to the left of an observer could be located to the right of an avatar (see Figure 3, right panel, overleaf). Thus, as with Experiment 1, it is reasonable to assume that the spontaneous perspective taking theory predicts that the effect this should occur in other paradigms in which the avatar either sees the same stimuli as the participant or does not. In the present experiment participants undertook a variant of a standard Simon task. Importantly, in half of the trials the stimulus to be discriminated appeared on the same side of the display with respect to both the participant and the avatar (e.g., left side for both). On the other half, the stimulus was on one side relative to the participant (e.g., left), but on the other side (e.g., right) relative to the avatar. The spontaneous perspective taking theory predicts that RTs will be shorter in the former condition relative to the latter because the position (and, thus, perspective) of the critical stimulus with respect to left and right is the same for the avatar and participant.

#### Experiment 2 Results

**Figure 4:**
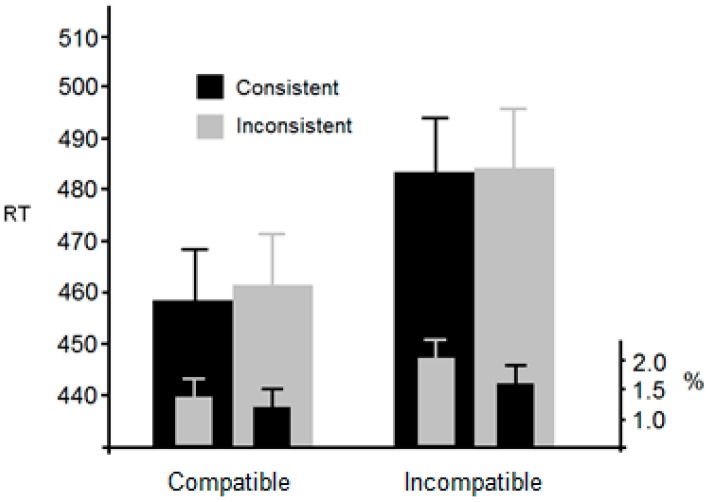
Mean RT and error rates from Experiment 2 together with standard errors. Compatible/Incompatible refers to the manipulation that generates the classic Simon effect.

Figure 4 presents mean RTs for each condition. Outliers (2 SDs; 4.1%) were again omitted from further analysis. An ANOVA with compatible and incompatible as within-participant factors revealed a significant main effect of compatilbility, *F*(1, 25) = 14.8, *p* < 0.001, η^2^_p_ = 0.37, but no significant main effect of consistency, *F*(1, 25) < 1. The interaction was not significant, *F*(1, 25) < 1.

Error data revealed a small, although non-significant, main effect of compatibility, *F*(1, 25) = 3.9, *p* < 0.06, η^2^_p_ = 0.13, and consistency, *F*(1, 25) = 3.3, *p* < 0.08, η^2^_p_ = 0.11. The interaction was not significant, *F*(1, 25) < 1. Overall, Experiment 2 has shown a classic Simon effect; RTs were reduced when the target was located on the same side as the response required. However, this effect was not influenced by the location of the target with respect to the avatar’s viewpoint. As with Experiment 1, this is not consistent with the hypothesis that observers spontaneously took the perspective of the avatar.

### 1.3. Experiment 3 Introduction

**Figure 5:**
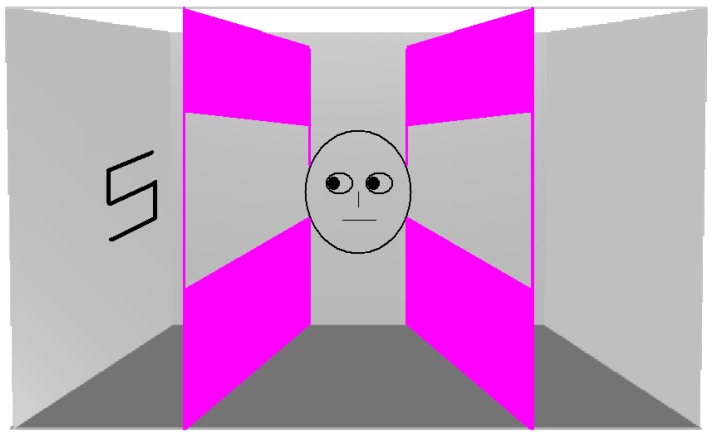
Example of a stimulus employed in Experiment 3. The figure shows a seeing condition valid trial.

The notion of spontaneous perspective taking has not only come from the dot-perspective task. Results from the classic gaze cueing paradigm have also been argued to be due to, or at least modulated by, visual perspective taking. For instance, Nuku and Bekkering [3] pointed out that previous studies had not examined whether gaze cueing occurs as “a consequence of *observing* the others’ gaze direction or a consequence of inferring the others’ attended location” (p. 340). By manipulating whether the gazing agent’s eyes were open or closed, the authors went on to show that the gaze cueing effect only occurs “where the agent is believed to be attending to the object” (p. 340). Thus, ‘believing’ what the agent can or cannot see is clearly invoking ToM processes. However, using the barrier technique described in the Introduction above, Cole et al. [8] showed that the gaze following effect still occurs when the gaze cue cannot see the targets. This was observed when the gazing agent was both a real, physically present, person who was sat opposite the participant and when it was a photograph of a person presented on a monitor. Given the contradictory findings of previous work, the present Experiment 3 again examined the visual perspective account, this time with specific reference to the gaze cueing effect. We employed a schematic representation of a face (Figure 5), typically employed in this paradigm, together with the barrier manipulation. In half of the trials, the gazing agent could see the two lateral walls and, hence, targets. However, on the other half, the window-like structures were blocked, thus, preventing visibibility of the walls. As previously, if the computation of the gazing agent’s visual perspective underlies the gaze cueing effect, no such effect should occur when the agent cannot see the targets. 

#### Experiment 3 Results

**Figure 6:**
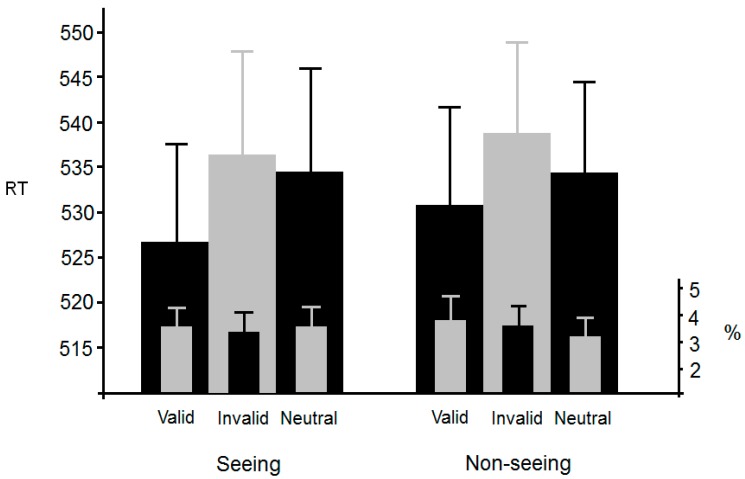
Mean RT and error rates in Experiment 3. Standard error bars are also shown.

Of the responses, 4.2% were outliers (2 SDs) and were omitted from further analysis. Figure 6 shows mean RTs for each of the six conditions. An ANOVA with validity (valid, invalid, or neutral) and visibility (seeing or non-seeing) as within-participants factors revealed a significant main effect of validity, *F*(2, 82) = 3.5, *p* < 0.05, η^2^_p_ = 0.078, but no significant main effect of visibility, *F*(1, 41) < 1. The interaction was not significant, *F*(2, 82) < 1. 

The first notable aspect of these results is the presence of an attention cueing effect. Participants were faster to identify the target when it appeared in the cued relative to uncued location. This replicates the many previous reports of eye gaze triggering a shift in an observer’s attention (e.g., [4,5]). Crucially however, is the finding that this effect was not influenced by what the gazing agent could see. That is, a gaze-following effect was observed even when the agent could not see the targets. As with Experiments 1 and 2 this does not support the spontaneous perspective taking theory. In sum, Experiments 1–3 manipulated consistency between what the participant and a gazing agent could see. Results have shown no modulation of a basic visual cognition phenomenon (i.e., the flanker effect; the Simon effect; gaze-following) based on whether a gazing agent could see the critical stimuli or not. In our final two experiments, we examine an alternative explanation for the results that have been attributed to automatic perspective taking.

### 1.4. Experiment 4 Introduction

**Figure 7:**
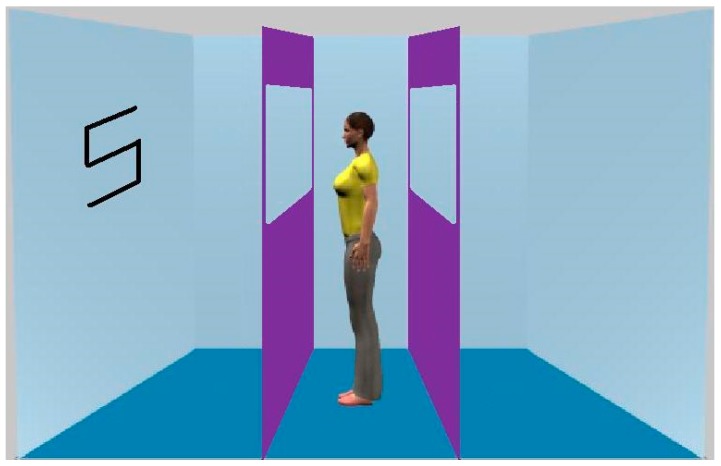
Stimuli used in Experiment 4. The example shows a seeing condition, valid trial.

Experiments 4 and 5 examined the claim of Samson et al. [1] and Santiesteban et al. [6] that avatar-induced shifts of attention (in the dot-perspective paradigm) contribute or, indeed, generate the consistency effect. Recall that, for Samson et al. [1], such a shift provides a mediating mechanism with which spontaneous perspective taking occurs, whereas for Santiesteban et al. [6] it is the explanation. In Experiment 4 we carried out a close replication of a standard central cueing experiment in which the avatar employed previously (i.e., the present Experiments 1 and 2; [1,6];) was used as the cueing stimulus. Thus, a single target appeared in either the looked-at direction or in the opposite hemifield (see Figure 7). As with Experiment 3, we again manipulated whether the avatar could see the lateral walls or not with the use of barriers. This manipulation enabled us to again examine the perspective taking theory, in addition to the attentional shift hypothesis, since no effect should occur when the avatar cannot see the target. Since the effects of central cues are thought to require some time to build up [14], we additionally employed a cue-target interval of 100 ms, allowing a relatively liberal test of the directional hypothesis. Note that this interval may still be too short to allow any shift to occur. For instance, Bukowski et al. [2] and Gardner et al. [15] showed that an interval of 300 ms or longer may be needed. However, intervals of this magnitude increase the likelihood of top-down proceeses modulating any effect. This, by definition, could mean that the phenomenon is not ‘automatic’ or ‘spontaneous’.

#### Experiment 4 Results

**Figure 8:**
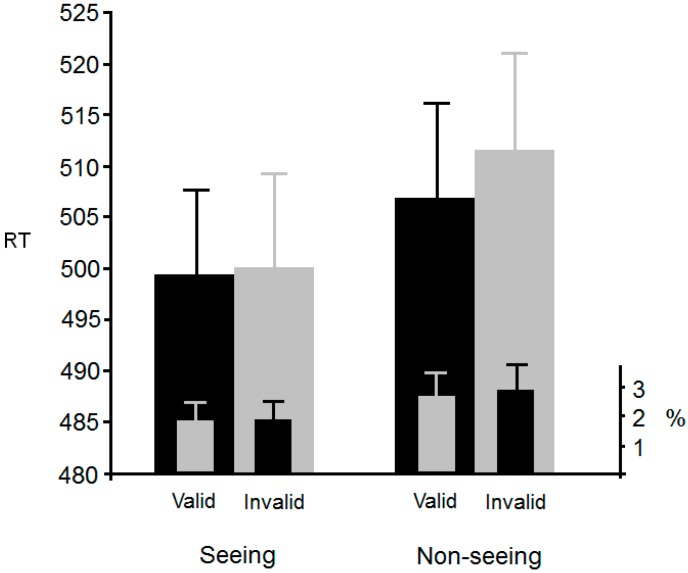
Mean RT and error rates from Experiment 4 together with standard errors.

Outliers (2 SDs) were removed, accounting for 4.1% of the data. One observer was removed from further analysis due to an error rate of more than 20%. Figure 8 (overleaf) shows mean RTs. An ANOVA with validity (valid or invalid) and visibility (seeing or non-seeing) as within-participant factors revealed no significant main effect of validity, *F*(1, 31) = 1.1, *p* > 0.3, or visibility, *F*(1, 31) = 2.2, *p* > 0.14. The interaction was also not significant, *F*(1, 31) = 0.59, *p* > 0.44. With respect to the error data, there was no significant main effects of validity, *F*(1, 31) = 0.7, *p* > 0.4, or visibility, *F*(1, 31) = 2.5, *p* > 0.11. The interaction was also not significant, *F*(1, 31) = 0.7, *p* > 0.38. 

Overall, the results from Experiment 4 reveal that the avatars employed in the present and previous works are not able to shift attention to the side. Furthermore, the absence of a cueing effect was apparent in both visibility conditions. Not only do these data fail to support the perspective taking account (i.e., RTs were not facilitated when the avatar could see the target), but they also fail to support the existence of a suggested mechanism [1,6] with which automatic perspective taking could occur, i.e., attentional cueing.

### 1.5. Experiment 5 Introduction

Although Experiment 4 did not provide evidence that the kind of avatar previously employed shifts attention, one could argue that this conclusion is weak because it rests on a null effect; perhaps our experimental setup was not sensitive enough to reveal an attentional shift if it exists. Furthermore, although Experiment 4 (and Experiment 3) included an avatar-target interval of 100 ms in an attempt to assist the generation of a cueing effect (see Methods below), one could argue that no such interval should be included since Samson et al. [1] and Santiesteban et al. [6] did not include one. Moreover, these authors did not include barriers in their displays. 

In our final experiment we directly compared the cueing ability of the avatar used previously with that of a stimulus known to induce attentional shifts. An abundance of work has demonstrated that a luminance change, and/or object onset, that occurs shortly before a target, is particularly effective at marshalling attention [14,16]. If our method is not sensitive enough to induce/measure attentional shifts we should not find a cueing effect for both the avatar and luminance cues. If, by contrast, we observe attentional cueing with at least one of our cues we can be confident that our paradigm is indeed sensitive to index such a shift.

#### Experiment 5 Results

**Figure 9:**
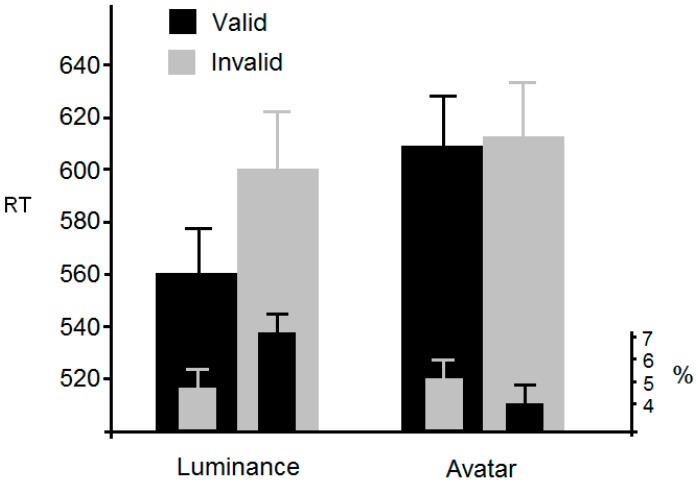
Mean RT and error rates from Experiment 5 together with standard errors.

Outliers (2 SDs) were removed accounting for 4.3% of the data. Figure 9 shows the mean RTs. An ANOVA with validity and cue-type as within-participant factors revealed a significant main effect of validity, *F*(1, 27) = 15.9, *p* < 0.001, η^2^_p_ = 0.37, and cue type, *F*(1, 27) = 22.6, *p* < 0.001, η^2^_p_ = 0.46. The interaction was also significant, *F*(1, 27) = 12.1, *p* < 0.002, η^2^_p_ = 0.31. Simple analyses revealed that the interaction was due to a cueing effect occurring in the luminance-cue condition, *t*(27) = 4.4, *p* < 0.001, but not in the avatar-cue condition, *t*(27) = 0.89, *p* > 0.38. With respect to errors, an ANOVA using the same factors and levels described above revealed a non-significant main effect of validity, *F*(1, 27) = 2.2, *p* < 0.16, η^2^_p_ = 0.08, and cue type, *F*(1, 27) = 2.8, *p* < 0.12, η^2^_p_ = 0.1. The interaction was, however, significant, *F*(1, 27) = 6.2, *p* < 0.02, η^2^_p_ = 0.19. Simple analyses revealed a significant reduction of errors in the luminance-cue condition, *t*(27) = 2.4, *p* < 0.05, but not in the avatar-cue condition, t(27) = 0.69, *p* > 0.49. 

Overall, these data confirm the results of many previous attentional orienting studies; the onset of a luminance cue is effective at marshalling attention. By contrast, the avatar was not able to shift attention, thus supporting the results of Experiment 4. This, in turn, shows that our procedure is sensitive enough to index any attentional orienting, if one exists.

## 2. General Discussion

A number of studies suggest that observers spontaneously compute the perspective of other individuals. This is based on experiments showing that responses are longer when a human agent, located in a visual display, does not see the stimuli that are relevant to an observer’s task. We assessed this theory by asking whether two classic visual cognition effects are also modulated by what an agent could see. In Experiment 1, we observed a robust flanker effect, but one that was not influenced by whether an avatar could see the relevant inducing stimuli or not. A similar effect was observed in Experiment 2 in which a Simon effect was not found to be influenced by an avatar’s vision of the stimuli that induces the compatibility phenomenon. Given previous contradictory findings, Experiment 3 reassessed the claim that perspective taking also underlies the gaze-cueing effect. We found that the effect was not modulated by whether the gaze cue could see the target or not. In Experiments 4 and 5 we examined the previous claim that results from the dot-perspective paradigm are due to the gazing agent’s capacity for shifting attention. The results found no evidence of this.

Overall, these data challenge the spontaneous perspective taking claim and a possible mechanism that could mediate it. At the very least they show that the phenomenon is not particularly robust since it is susceptible to small changes in the stimuli and/or task (i.e., Experiments 1 and 2). That is, the effect may not easily generalize beyond the dot-perspective task. Our findings, therefore, support other recent work by Gardner, et al. [15] who also examined the attentional shift hypothesis. They too found that the avatars employed by Samson et al., were not able to (rapidly) shift attention. However, interestingly, and perhaps most significantly, Gardner et al. did find a significant cueing effect when the targets appeared 600 ms after the avatar. This, in turn, suggests that the cueing effect is under top-down control; at 600 ms post-avatar presentation, participants will begin to have time to consider what the avatar is facing towards, that is, non-automatically. Further evidence for the possible non-automatic nature of the basic perspective taking phenomenon also comes from an additional experiment of Gardner et al. The authors replicated the dot-perspective procedure with the exception that participants were never told that the experiment concerned ‘perspective taking’ and they were never required to consider the avatar’s perspective. Thus, participants were unlikely to have adopted a top-down set for ‘perspective taking’. Results showed that no consistency, i.e., perspective taking, effect occurred. These data, and the current findings, thus represent a growing challenge to the automatic perspective taking theory (see also the present General Introduction and [17]). 

This challenge does however have to be placed against other recent work showing that gazing agent-induced effects are modulated by what the agent can see. For instance, employing the barrier technique, Baker, Levin, and Saylor [18] placed an avatar to one side of a display (i.e., not in the centre) that always looked towards the middle. This ensured that its gaze direction was not confounded with a potential shift of attention because it always faced the same way. Unlike the present findings, and that of Cole et al. [7,8], Lewis et al. [9] did observe a perspective taking effect that was modulated according to what the avatar could see. Furthermore, Zhao, Cusimano, and Malle [19] have found evidence for spontaneous perspective taking using a very different task to that of the dot-perspective method. In their procedure (see also [20]), observers were presented with a photograph of a person sitting at a table facing the observer but looking down at a number placed on the table. The number was ‘6’ from the perspective of the person and ‘9’ from the perspective of the observer. Observers were simply asked to indicate what number was placed on the table. Although the number was ‘9’ from their own perspective, 42% of observers judged the number from the viewpoint of the person in the display (i.e., indicated ‘6’), suggestive of spontaneous perspective taking. Thus, with the exception of the Zhao et al. [19] data, the theory of spontaneous perspective taking is currently in the position where a number of very similar experiments are showing very different results.

One potential challenge to the present conclusion (i.e., no spontaneous perspective taking) concerns the distinction between ‘Level 1’ and ‘Level 2’ perspective taking [21]. The former is characterised as *whether* another person can see a stimulus and the latter is characterised as *how* another person sees the stimulus (e.g., its orientation). Surtees, Samson, and Apperly [20] provided evidence that participants are only able to spontaneously or ‘unintentionally’ compute an avatar’s perspective in terms of whether a stimulus can be seen, not how it is seen, i.e., Level 1 perspective taking. One could argue that, with the exception of Experiment 2, the present experiments only examined (the more stringent) Level 2 perspective taking because our targets were letters. Perhaps participants automatically computed that the avatars could see the letters but not how they were seen, that is, their identity. However, apart from the fact that no such cognitively demanding processing of the discs was required in Experiment 2, we raise the possibility that visual perspective taking can only occur in the Level 2 sense. To take another person’s visual perspective has to mean representing how they see a stimulus. It is something of a contradiction to suggest that the perspective of another person can be taken without doing so. Indeed, if an actual perspective is being taken, that is, a percept is represented, what exactly is being seen if not how something looks? This is no different to asking how a person could possibly have *their own* perspective of an object and yet not see or know how the object looks to themselves. We suggest that this necessarily awkward circularity is no different to the notion of taking another person’s perspective. Of course, an observer *can* know that another person sees a stimulus without knowing how it is seen to them, i.e., ‘Level 1 perspective taking’ does exist. However, this does not need to be based on taking anothers’ actual perspective; it can occur via the mental drawing of a straight line between the eyes of the person being observed and any particular object in the scene. If the straight line is unobstructed, the person can see the object. Furthermore, as the first to make the Level 1–Level 2 distinction, Flavell and collegues often discussed the former in terms of ‘understanding’ and ‘knowledge’. The latter, by contrast, is more often discussed with reference to such notions as ‘visual experience’, ‘perspectival views’, and ‘perspective-derived’ [21].

## 3. Conclusions

In sum, it has been shown that the ‘perspective taking’ effect observed in the dot-perspective task does not generalise to other paradigms. A conservative interpretation of this result is that perspective taking effects can only be observed under highly constrained experimental settings. More broadly, the present work does not support the theory that humans spontaneously take the perspective of others. Although refuting a hypothesis, (e.g., the barrier technique) is more powerful than providing confirmatory evidence for it, future workers may want to examine why the dot-perspective effect occurs at all.

## 4. Materials and Methods 

For every experiment in this paper all participants gave informed consent before participating and each study was approved by the Essex University Department of Psychology Ethics Committee (GC1701).

### 4.1. Experiment 1

#### 4.1.1. Participants

A different set of participants was used in all the present experiments. In Experiment 1, there were 26 participants who took part in exchange for course credit.

#### 4.1.2. Stimuli and Apparatus

The virtual room was 19.8° wide and 12° high. A male or female human avatar (7.8° in height) was located in the centre and always faced to the left or right-hand wall. The room was shorter than that used by Samson et al. in order to make the flanking letter less peripheral and, hence, visible. Barriers were located to the left and right of the avatar and were approximately the same height as the room. The barriers had a section cut out, allowing the walls to be visible. Although these barriers were not relevant to the present task, we included them in order to have stimulus parity with our other experiments in which they were used to manipulate what the avatar could see. The target was either a letter ‘S’ or ‘H’ (both measuring 1.1° wide and 1.4° high) and would occur on the shoulder of the avatar. A single distracting letter appeared on either the left or righthand wall such that its centre was 5.2° from the fixation point. As with the target the distractor could either be an ‘S’ or ‘H’ and would occur on the left and right wall with equal frequency. These distracters were 5.5° high (measured along their middle) and 3.0° wide. On 50% of trials the target and distractor were the same (e.g., both were the letter ‘S’) whilst on the other 50% each were different. Thus, the target and distractor were either congruent or incongruent with each other. On half of the trials, the avatar faced towards the distractor whilst on the other half the avatar faced the opposite wall. Therefore, the avatar’s perspective of the distractor was either consistent with the participant’s or inconsistent. The room and barriers together with a black fixation cross were present as background throughout the entire experiment. As with Samson et al., male observers were presented with a male avatar and female observers were presented with a female avatar. The experiment was run on an Apple eMac (Apple, Cupertino, CA, USA) computer linked to a Cathode Ray Tube monitor.

#### 4.1.3. Design and Procedure

A within-participant, 2 × 2 factorial design was employed. The first factor manipulated whether the distractor was congruent or incongruent with the target. The second factor manipulated the avatar’s view of the distractor and, thus, the consistency of its own viewpoint with the participant (consistent, inconsistent). Each trial presented the avatar, target and distractor simultaneously. These offset when the participant responded and 1000 ms elapsed before the next trial. The participants were required to press a left-hand button if the target was an ‘S’ and a right-hand button if the target was an ‘H’. They were told to ignore the peripheral distractor. Observers were seated approximately 70 cm from the display and asked to make a response as quickly as possible whilst keeping errors to a minimum. Participants were shown an example of the stimuli and explicitly told that the avatar could either see the distractor or not depending of which way it faced. There were 192 trials in total, equally divided across all trial types. Twenty-four practice trials were given. All conditions of the experiment were presented in a single block.

### 4.2. Experiment 2

#### 4.2.1. Participants

There were 26 participants who took part in exchange for course credit.

#### 4.2.2. Stimuli and Apparatus

All aspects of these were as described previously with the following exceptions. A single red or green disc would appear on either the left hand or right hand wall of the room at the positions shown in Figure 3. A cross was located on both walls showing where the avatar gazed, and the avatar always faced the discs. The dimensions of the room were identical to those used by Samson et al. [1].

#### 4.2.3. Design and Procedure

A within-participant, 2 × 2 factorial design was employed. The first factor manipulated compatibility of the stimulus location and response location with respect to the participant (compatible, incompatible), i.e., the manipulation that generates the basic Simon effect. The second factor manipulated consistency of the participant and avatar viewpoints with respect to the location of the target. A consistent trial was one in which the target was on the same side of the display for both the participant and avatar (e.g., left for the participant, left for the avatar) and an inconsistent trial was one in which they were on different sides (e.g., left for the participant, right for the avatar). The participants were required to press a left-hand button if a green disc appeared and a right-hand button if a red disc appeared. All other aspects of the procedure were as described previously. Thus, there were 192 trials in total, equally divided across all trial types and presented in a single block.

### 4.3. Experiment 3

#### 4.3.1. Participants

Forty-two participants from the University of Essex took part in exchange for course credit. 

#### 4.3.2. Stimuli and Apparatus

The gaze cueing agent was a black schematic face measuring approximately 3.4° in height and 2.8° wide presented (see Figure 5). The targets were black letters, ‘S’ and ‘H’ (1.9° high, 1.4° wide), that could appear on the left or right. We manipulated the agent’s visibility of the targets with the use of the barriers described previously. On ‘seeing’ trials the barriers had a section cut out allowing the target to be seen. On ‘non-seeing’ trials, no such window-like structure was included. To ensure that the size of the cueing face was approximate to those typically used by previous gaze cueing studies, the barriers were positioned slightly further apart than were positioned previously. Furthermore, in an effort to increase saliency of the barriers, and hence knowledge of what the gaze cue could see, we made the room greyscale. The experiment was run on an Apple eMac computer linked to a CRT monitor.

#### 4.3.3. Design and Procedure

A within-participant, 3 × 2 factorial design was used. The first factor manipulated cue ‘validity’. The gaze cue either looked to the location of the target (‘valid’) or to the opposite side (‘invalid’). As with many gaze cueing experiments, neutral cues were included in which the face gazes straight ahead. The second factor manipulated the agent’s ability to see the room’s left and right hand walls and thus the target. The virtual room acted as the background for the experiment and each trial began with the presentation of the barriers for 1000 ms. This was followed by the onset of the central face which appeared 100 msec before the target joined it. Participants were asked to respond as quickly as possible when they identified the target letter. The target letter ‘S’ required a left button response and the target letter ‘H’ required a right button response. Forty-eight valid, 48 invalid, and 48 neutral trials were presented in both visibility conditions, thus generating 288 trials in total. The numbers of the different trial types were balanced such that there were an equal number of target types, target locations, and visibility conditions. This meant that the faces validly cued the target location on 33% of trials, with a further 33% being invalid and the remaining 33% being neutral. Twenty-four practice trials were given following the demonstration trial. All conditions of the experiment were presented in a single block.

### 4.4. Experiment 4

#### 4.4.1. Participants

There were 32 participants recruited from the University of Essex.

#### 4.4.2. Stimuli and Apparatus

All aspects of these were as described previously, with the exception that the barriers were located closer together than previously and the target was slightly more peripheral (see Figure 7).

#### 4.4.3. Design and Procedure

A within-participant, 2 × 2 factorial design was used. The first factor manipulated cue validity (valid, invalid). The second factor manipulated the avatar’s view to the lateral walls and, hence, targets, by using the barriers we employed previously. The room, with barriers and fixation point, acted as background and was thus present for the entire experiment. Each trial began with the presentation of the avatar for 100 ms followed by the target. The visibility condition was blocked and the presentation order counterbalanced. This blocked design was included so that attribution of what the avatar could see did not need to be computed trial-by-trial; again, a more liberal test of perspective attribution. Participants were shown an example of each type of barrier at the beginning of each visibility block and told that the avatar could either see or not see the two walls and targets. One hundred and ninety-two trials (i.e., 96 per visibility block) were presented equally divided amongst all trial types. Thus, the avatar cued the target location on 50% of trials. As previously, male observers were presented with male avatars and female observers were presented with female avatars. All other aspects of the design and procedure were as already described.

### 4.5. Experiment 5

#### 4.5.1. Participants

There were 28 participants recruited from the University of Essex.

#### 4.5.2. Stimuli and Apparatus

**Figure 10:**
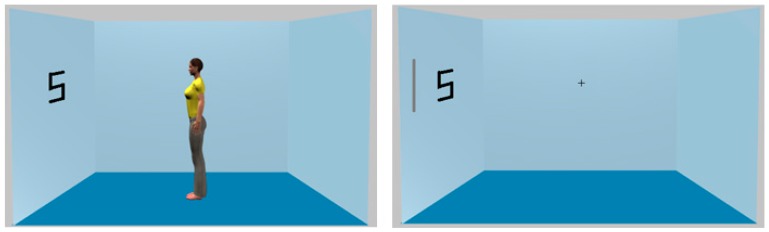
Examples of the stimuli used in Experiment 5. In the left panel the cue is the (‘valid’) avatar facing the target. In the right panel, the cue is an object onset (i.e., the grey line) that appears adjacent to the target.

All aspects of these were similar to those described previously with the following exceptions. The inducing stimuli in the avatar condition were identical to those employed by Samson et al. [1]. That is, the images were not changed in any way with the exception that a target letter (slightly smaller, but thicker than we used in Experiment 4) was located to the left or right in the position shown in Figure 10. The stimuli for the luminance cue condition were the same with the exception that no avatar was present and a grey line was presented on the left or right adjacent to where the target would appear (also shown in Figure 10). A fixation cross was also present in the luminance cue condition.

#### 4.5.3. Design and Procedure

A within-participant, 2 × 2 factorial design was again used. The first factor manipulated cue validity (valid, invalid) and second manipulated cue type (avatar, luminance). In the avatar cue condition, the avatar appeared simultaneously with the target. In the luminance cue condition, the cue appeared 100 ms before the target. As previously, the virtual room acted as background and was present throughout. All trial types occurred in a single block of 192 trials equally divided amongst all trial types.
